# High-Value Chemicals from Electrocatalytic Depolymerization of Lignin: Challenges and Opportunities

**DOI:** 10.3390/ijms23073767

**Published:** 2022-03-29

**Authors:** Rabia Ayub, Ahmad Raheel

**Affiliations:** 1 RISE Processum AB, Bioeconomy and Health Division, SE-891 22 Örnsköldsvik, Sweden; 2 Department of Chemistry, Quaid-i-Azam University, Islamabad 45320, Pakistan; ahmadraheel001@gmail.com

**Keywords:** lignin, electrocatalysis, depolymerization, valorization, high-value chemicals

## Abstract

Lignocellulosic biomass is renewable and one of the most abundant sources for the production of high-value chemicals, materials, and fuels. It is of immense importance to develop new efficient technologies for the industrial production of chemicals by utilizing renewable resources. Lignocellulosic biomass can potentially replace fossil-based chemistries. The production of fuel and chemicals from lignin powered by renewable electricity under ambient temperatures and pressures enables a more sustainable way to obtain high-value chemicals. More specifically, in a sustainable biorefinery, it is essential to valorize lignin to enhance biomass transformation technology and increase the overall economy of the process. Strategies regarding electrocatalytic approaches as a way to valorize or depolymerize lignin have attracted significant interest from growing scientific communities over the recent decades. This review presents a comprehensive overview of the electrocatalytic methods for depolymerization of lignocellulosic biomass with an emphasis on untargeted depolymerization as well as the selective and targeted mild synthesis of high-value chemicals. Electrocatalytic cleavage of model compounds and further electrochemical upgrading of bio-oils are discussed. Finally, some insights into current challenges and limitations associated with this approach are also summarized.

## 1. Introduction

There is a strong need for sustainable alternatives for the production of building block, fine, and commodity chemicals due to limited and continuously depleting fossil resources and increased environmental crises. To address these challenges, the generation of bioenergy and biochemicals obtained from biomass can be an alternative [1]. Electrocatalysis, a promising energy-efficient technology, has gained significant interest in renewable feedstocks, especially in lignin, the most attractive alternative source of commodity chemicals in nature [2]. Electrocatalysis is a promising technology that can be used for energy renewal, storage of energy in terms of chemicals, and for environmental purification [3,4]. Electrochemical methods have several advantages, i.e., highly sustainable, environmental friendliness, high selectivity, precisely controlled lignin degradation, and simultaneous hydrogen production in case of electrochemical oxidation and simultaneous production of oxygen when electrochemical reduction occurs [5]. These methods do not require complicated organometallic catalysts in comparison to homogeneous and heterogeneous catalysis. These electrocatalytic processes are less energy-intensive in comparison to hydrothermal liquefaction, gasification, and pyrolysis. Furthermore, proper control of electrode voltage, composition, and applied current density can generate intermediates and final products in a good selectivity. Oxidation of lignin can be carried out in slightly basic media using an electron transfer mechanism.

Lignin is a heterogeneous three-dimensional polyphenolic macromolecule that is non-uniformly linked by different aryl–aryl, aryl–alkyl, alkyl–alkyl, and aryl–aryl ether groups leading to diphenylether linkage, dibenzylether linkage, β-O-4, β-5, β-β, and β-1 linkages. The most abundant of these linkages is the β-O-4 linkage which comprised 50% in spruce and 60% in eucalyptus and birch linkages [6]. Lignin is a potential source of many renewable chemicals, vanillin, vanillic acid, dispersing agents, ion-exchange resins, activated carbons, polymer fillers, fuels, and tailor-made materials [7,8,9,10,11,12]. A large amount of lignin is produced from the paper and pulp industry during the pulping process as a by-product. Most of this lignin is used as a low-value thermal energy source besides its promising applications as carbon building blocks [13]. To date, many approaches to catalytic oxidations and reductions, [14] including thermal, [15] biological, [16] homogeneous, and heterogeneous catalysis [17] have been used to valorize and depolymerize lignin. However, the main limitations of these approaches are poor selectivity, low yields of depolymerized products, high price and toxicity of oxidizing and reducing agents, and formation of char-like products, making them difficult to scale up and commercialize [18,19,20]. Efforts have been made to develop novel and attractive strategies for the full valorization of lignin. In the last 20 to 30 years, electrochemical methods have started gaining increased interest in the biomass research field. Two kinds of approaches have been used so far, untargeted electrochemical lignin degradation and targeted lignin electrochemical degradation leading to specific product formation [21,22]. However, significantly less attention is given to targeted lignin depolymerization technology so far. Untargeted lignin degradation often gives a mixture of different products with various functionalities which requires a range of different analytical techniques, e.g., HPLC, GCMS, SEC, FTIR, NMR, etc., to identify the obtained products [23].

In this regard, the first study on electrochemical depolymerization of lignin dates back to the 1940s when Bailey and Brooks reported oxidation and reduction of four different lignin types in 1% NaOH solution using different metal electrodes at various current densities (1–3 A/dm^2^) [24]. However, longer reaction times (12–96 h) were reported in their study. Moreover, mercury and lead electrodes gave good results. The highest yields of the monomeric compound were obtained when butanol-based organosolv lignin was used. After that, the research area was dormant for almost 30 years, and then for the last 20–30 years this research area has gained a lot of significant interest. Stephenson and co-workers reviewed various methods for lignin oxidation where enzymes and homogeneous and heterogeneous catalysts have been utilized [25]. We divided this review into two parts, the first part describes untargeted lignin depolymerization while the second part is focused on the targeted synthesis of valuable products obtained after electrocatalysis. We also discuss in brief what has been reported earlier on electrochemical degradation and upgrading of lignin model compounds.

## 2. Mechanism in Brief

**Scheme 1:**
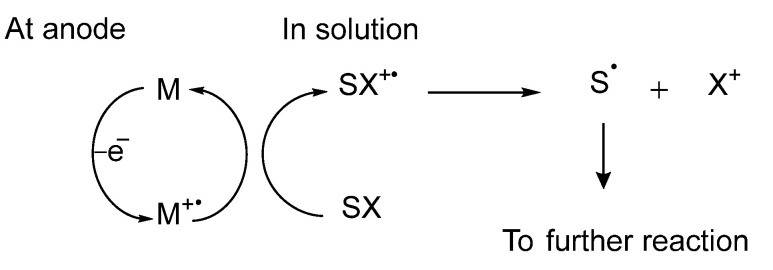
Indirect electrocatalysis. M is metal electrode while SX is substrate.

Before further going into the details of different electrochemical methods, we will briefly describe mechanism. An electrochemical reaction can take place either in a direct or an indirect manner. In indirect electrocatalysis, an electrocatalyst or a redox mediator can promote electron transfer between electrode and lignin molecules. It involves heterogeneous electron transfer reactions and homogeneous redox reactions. A redox mediator reacts with a substrate molecule through a homogeneous reaction and is regenerated at the electrode surface after transferring electrons. A redox mediator must be stable in its oxidized and reduced forms and should not undergo any cleavages (Scheme 1).

For indirect electrolysis, when water is used as a reaction media in oxidation reactions, for example, water is electrolyzed to hydroxyl radicals and protons. Oxidation can take place directly from hydroxyl radicals or these hydroxyl radicals can chemisorb on the electrode surface (anode) and catalyze the reaction [26]. These chemisorbed hydroxyl radicals convert to higher metal oxides MO which can catalyze the oxidation reaction (Equations (1)–(3)) [27,28]. While in the course of direct electrocatalysis, no extra catalyst is required and only the electrode can transfer electrons to the substrate molecule.

(1) $$M+H_{2}O\;\to \;\;\;M\left(\dot{O}H\right)+H^{+}+e^{-}\;\;\;$$

(2) $$M\left(\dot{O}H\right)\;\;\to \;MO+H^{+}+e^{-}\;\;$$

(3) MO+R  →   M+RO  

## 3. Untargeted Approach/Electrocatalytic Conversion of Lignin

### 3.1. Oxidative Approach

Untargeted oxidation of lignin is mostly reported to increase the carboxylic group content and decrease the phenyl content of the lignin [29]. Extreme oxidation conditions could lead to ring opening of the benzenic rings which resulted in less phenyl group content. The presence of the oxygen and electron carrier K_4_Fe(CN)_6_ can significantly lower the anodic deposition when anodic oxidation was performed at the Pt anode in the presence of oxygen/nitrogen.

Interestingly, electrochemical oxidation of primary alcohol groups leading to the following carboxylic acids in lignin was achieved in moderate to high yield and high selectivity when Stahl and co-workers used 4-AcNH-TEMPO in the presence of a NaHCO_3_/Na_2_CO_3_ buffer (pH:10) and H_2_O-MeCN solvent mixture [30]. They observed that the chemoselectivity of the reaction can be controlled by the acidity or basicity of the reaction media. Primary alcohol oxidation products are obtained under a basic environment while an acidic environment promotes secondary alcohol oxidation. Furthermore, the lignin used in their study was extracted from poplar wood and had a high content of β-O-4 linkage. The obtained oxidized lignin was highly soluble in water which makes it an excellent candidate for polymer-derived applications. However, the oxidized lignin was further susceptible to depolymerization to the aromatic monomers in 30 wt% yields under acidic conditions.

Moreover, a combined one-pot, two-step electrochemical oxidation-photochemical cleavage of native lignin at room temperature was demonstrated by Stephenson and co-workers [31]. In their investigation, electrochemical oxidation was mediated by PINO which promoted selective benzylic oxidation in high yields. Pine lignin and β-O-4 lignin model substrates were evaluated in their study. They observed the formation of aromatic ketones and phenols after crude electrochemical oxidation mixture was directly fed to the continuous-flow reactor for photochemical β-O-4 bond cleavage in the presence of iridium photocatalyst [Ir(ppy)_2_[dtbbpy)] (PF_6_).

In a non-diaphragm electrolytic cell consisting of graphite felt cathode and a RuO_2_-IrO_2_/Ti anode, anodic oxidation of lignin in alkaline media was reported by Zhu and co-workers [32]. They claimed that lignin can be oxidized either directly by anodic oxidation or from cathodically generated H_2_O_2_. H_2_O_2_ was generated on the cathode from O_2_ and this H_2_O_2_ was further decomposed to ROS which increased the efficiency of lignin oxidation. The formation of 20 different products was observed by GCMS and ESI-MS. Higher yields of the low molecular weight (LMW) products were obtained with an increased current density that promoted higher production of H_2_O_2_ and ROS species, while the increased temperature assisted the decomposition of H_2_O_2_. Finally, they claimed that the supply of additional external oxygen increased the depolymerization of lignin to 59.2% after 1 h of electrolysis at 80 °C with 8 mA/cm^2^ of current density.

Similarly, in another study, an integrated approach of anodic oxidation together with cathodically generated H_2_O_2_ mediated oxidation is reported in an undivided cylindrical electrolytic cell where a graphite felt anode was inside and RuO_2_-IrO_2_/Ti mesh on the outer side was used as a cathode [33]. Twenty-two kinds of different LMW products including monomers and dimers were observed by GCMS as a result of C–C and C–O bond cleavages. This integrated approach resulted in higher depolymerization efficiency of lignin.

Moreover, nitrobenzene-mediated anodic oxidation of organosolv spruce lignin is found to be taken place at slightly lower temperatures (170 °C) in comparison to the regular thermal nitrobenzene oxidation process [34]. The electrolysis proceeded at 4 mA/cm^2^ at 1.8–2.0 V for 4 h to produce depolymerized products (0.36–30% yield), mainly vanillin, phenol, and 4-hydroxybenzaldehydes as major products. In some cases, alkaline pretreatment enhanced the reaction efficiency and promoted an anodic oxidation mechanism via the formation of epoxides. It was proposed that nitroaromatics acted as electron transfer oxidants and gets reduced themselves. Furthermore,1,3-dinitrobenzene was found to be a more efficient mediator than nitrobenzene.

Using a statistical design of experiment (DoE) approach, Stiefel and co-workers studied the effect of five different parameters (temperature, alkalinity, electrode material, lignin concentration, and current density) on technical lignin depolymerization and solubility of obtained lignin in an acidic solution [35]. Their results showed that depolymerization efficiency is independent of sodium hydroxide concentration. Acid solubility of obtained lignin did not change significantly when the temperature was increased from 30 to 80 °C. However, a high degree of depolymerization and increased monomer production were achieved with increased current density. A decreased lignin concentration generated an increased number of monomeric products (vanillin). With regard to the electrode material, a lower number of monomeric products was obtained at the Pt electrode in comparison to Ni. According to their results, optimal depolymerization of technical lignin was obtained at 1.8 A on the Ni electrode at 30 °C when a 5 g/L lignin concentration was used. Vanillin and vanillic acid were obtained in 61–67 and 23–30% yields, respectively. Finally, no correlation between any of the lignin properties, e.g., acid solubility vs. molecular weight, molecular weight vs. monomer yield, and UV absorbance vs. molecular weight was observed.

Electrochemically reduced (EC) TiO_2_ nanotube arrays prepared by anodization of a Ti metal plate were evaluated for electrochemical oxidation of lignin and a linear correlation was observed between the length of the TiO_2_ nanotube array and electrocatalytic efficiency of lignin oxidation [36]. Oxidative degradation of lignin was performed in a three-electrode cell using a 0.1 M NaOH solution with 100 ppm of lignin concentration. The TiO_2_ electrodes of different lengths (4.2–15.5 μm) fabricated by anodization under different reaction times (4–20 h) were examined and high electrocatalytic efficiency was observed from 13.5 μm TiO_2_ nanotubes prepared after 16 h. A higher current density of 6.58 mA/cm^2^ (330 times) was observed at 1.9 V in comparison to the untreated TiO_2_ electrode (0.02 mA/cm^2^). It was observed that the 16 h anodized TiO_2_ nanotube arrays even perform eleven times better than the Pt electrode. Moreover, 70% of total organic carbon (TOC) content was observed after 3 h of electrocatalytic oxidation, and an increase in temperature to 30 °C increased the oxidation efficiency. Efficient lignin degradation was observed at 4 mA/cm^2^ from 16 anodized electrode materials and a further increase in the current reduced the rate of reaction which might be due to increased oxygen evolution at higher current density. The electrochemically reduced TiO_2_ nanotube electrode was found to be stable even after eight oxidation cycles of each 24 h reaction time.

Moreover, lignosulfonate when electrocatalytically degraded onto PbO_2_ membrane electrode exhibited lower surface tension and oil-water interfacial tension in comparison to raw lignosulfonate [37]. Several fractions with low molecular weight lignin were obtained after electrocatalytic degradation. However slight condensation to larger molecular weight fractions was also observed. The effects of reaction conditions were also evaluated on the β-O-4 lignin model compound (1-(3-methoxy-4-hydroxyphenyl)-2-(2-methoxyphenoxy)ethanol) under similar reaction conditions. Production of degraded products was mainly affected by pH, current density, voltage, and electricity consumption. It was found that phenolic hydroxyl, carboxyl, and aldehyde functionality increased in the beginning, and continued electrolysis reaction time decreased their content significantly while no effect on the sulfonic group was observed. This behavior could be due to cleavage of aryl–ether linkage which increased the hydroxyl content and further oxidation decreased the total amount of phenolic hydroxyl. A decrease in the carboxyl group could be due to the cleavage of carbon–carbon bonds while the sulfonic group was harder to remove therefore no substantial effect on their content was observed. Effective degradation was observed at 2.5–3.0 V and it was observed that a minimum of 2.0 V was compulsory while voltage higher than 3.0 V resulted in ring cleavage. A current density greater than 10 mA/cm^2^ was found to degrade the PbO_2_ electrode and removed the electrode from the membrane surface. The substantial effect of pH changes was also observed as too low pH resulted in corrosion of electrodes and precipitation of lignosulfonate.

Similarly, electrocatalytic oxidation of lignosulfonate solution in the presence of sodium sulfate and sodium chloride in a single-cell flow electrochemical reactor is reported to remove total organic carbon in 80% yield at a current density of 30–60 mA/cm^2^ [38]. A single-cell flow electrochemical reactor with a boron-doped diamond electrode was employed to investigate the technical applicability of the process. A kinetic model was also developed to study the impact of current density on the kinetic parameters, for example, mass transfer coefficients and electrochemical reaction kinetic constants. It was found that reaction efficiency and reaction kinetics were mainly related to the current density and initial amount of chemical oxygen demand by a hyperbolic equation which showed first-order surface electrochemical reaction kinetic constant and a mass transfer coefficient, while the reaction progress was independent of the amount of sodium chloride/sodium sulfate. A 25 g/L lignin concentration was used. Furthermore, supporting electrolytes did not influence the reaction kinetics and had no effect on the TOC removal. It was assumed that organic acids, oxalic, and maleic acid might form before they were finally converted to CO_2_.

Furthermore, electrocatalytic depolymerization of kraft lignin over 3D-structured nickel electrode materials followed by subsequent continuous membrane filtration is reported to result in significant improvement in the product yields [39]. The reaction was performed at ambient pressure and room temperature and monomeric products together with significant reduction of molecular weight (93% to 220 Da) of the residual lignin were obtained after 4 h of electrolysis time. Continuous membrane separation increased the yield of monomeric products three times in comparison to without any separation. It was also observed that depolymerization increased with an increase in the electrode area. Moreover, the morphology of the electrode also affected the depolymerization and energy dependency of the process. Efficient depolymerization was observed from foam stacked electrodes followed by unstructured fleece and single foam electrodes. Only 2 A of current was sufficient to perform depolymerization on foam stack electrode in comparison to fleece electrode which required 8 A in order to give a similar result to that of 2 A on foam electrode.

The conversion of lignin into value-added aromatic compounds was carried out through its electrochemical oxidation by the use of β-PbO_2_/MWNTs [40]. These β-PbO_2_/MWNTs acted as electrochemical catalysts and are prepared by deposition of β-PbO_2_ (prepared from the hydrolysis of lead(IV) cetate) on MWNTs in acidic media. These catalysts were prepared in different formulations of 25% β-PbO_2_, 33% β-PbO_2_, 50% β-PbO_2_, and 75% β-PbO_2_. The electrochemical oxidation of the lignin was carried out with the help of these electrocatalysts, and the products obtained after the depolymerization were identified with the help of GCMS. It was found that these catalysts increased the rate of lignin depolymerization at low over-potentialand increased the process efficiency. It was found that 33% β-PbO_2_ gave the highest rate of depolymerization. Oxidative products and their concentrations were estimated after 24 and 48 h at a constant potential of 0.5 vs. SHE. Vanillin, methyl salicylate, 1,3-bis(1,1-dimethylethyl)-benzene, and 2,4-bis(1,1-dimethylethyl)-phenol are the major oxidation products identified by GCMS. One limitation was also observed for these catalysts, which was a reduction in their activity with the passage of time owing to the surface poisoning of the electrocatalysts. However, overall these β-PbO_2_/MWNTs electrocatalysts showed a high rate of low energy electrochemical conversions.

Movil and co-workers have synthesized nonprecious metal alloy nanoparticle electrocatalysts (Ni/C, Co/C, and NiCo/C electrocatalysts) via simple solution-based procedures [41]. These non-precious metal alloy nanoparticle electrocatalysts were used for electrochemical modification of lignin in alkaline solution at lower potentials than required for oxygen evolution. It was found that lignin oxidation was quasi-reversible or irreversible which formed oxidation products that likely further reacted via homogeneous chemical reactions or additional heterogeneous charge transfer steps. These results demonstrated that lignin oxidation (at lower over-potentials than required for oxygen evolution) leads to the efficient generation of hydrogen. Moreover, the oxidation of nonprecious metal electrocatalysts was also reported, with reversible behavior due to the oxidation/reduction process of the electrocatalysts. These nanoparticle electrocatalysts were found to increase the accessible surface area for electrochemical reactions, which might lead to enhanced mass transport of reactants and products across the electrocatalyst layer. The catalytic activity results showed that Co/C has the greatest rate of lignin oxidation, whereas Ni/C and NiCo/C have equal rates. FTIR and UV-VIS spectroscopy studies validated the electrochemical oxidation of lignin. Furthermore, the complexity of the process yielding several potential products and chain reaction pathways, and comprising proceeding homogeneous chemical reactions with rapid kinetics, prevented the establishment of a lignin oxidation mechanism.

In another study, Yan and co-workers described a simple and successful electrochemical method for depolymerizing three native lignin samples extracted from ethanol organosolv pretreatment of various biomass, including sweetgum, aspen, and pine [42]. The findings showed that low-cost, commercially available nickel foam could be used directly as a working electrode for the oxidative depolymerization of lignin samples. To examine and evaluate the results of electrolysis, gas chromatography-mass spectrometry, (2D) heteronuclear single quantum coherence nuclear magnetic resonance (NMR) spectroscopy, and gel permeation chromatography were used. The primary small molecule products of electrochemical depolymerization of aspen organosolv and sweetgum lignin electrolysis were determined to be vanillin and syringaldehyde (17.5% highest combined yield of vanillin and syringaldehyde). There was also no syringaldehyde identified in the electrolysis of pine lignin. Furthermore, the authors proposed that using hierarchically porous nickel-based electrocatalyst coated on nickel foam, rather than merely a nickel foam electrode, increased the performance of electrochemical depolymerization.

Wang and co-workers studied a novel approach for electrochemical catalytic degradation of aspen lignin in a three-dimensional electrode (TDE) reactor with a Pb/PbO_2_ anode (in alkaline medium) [43]. The surface morphology, composition, and electrochemical redox capability of the synthesized Pb/PbO_2_ electrodes were studied using SEM, XRD, and cyclic voltammetry. The gas chromatography revealed the presence of 4-methylanisole and some additional co-products (such as vanillin, syringaldehyde, 2,6-dimethoxyphenol, acetovanillone, toluene, and styrene) in the oxidative degradation of lignin. The β-PbO_2_ crystal on the surface of the Pb/PbO_2_ electrode was successful in delivering OH radical for lignin oxidative breakdown, while the [H] atom was provided by water molecule electrolysis as the cathode potential went lower than the equilibrium potential. Lignin was first broken into intermediates by OH radical oxidation, and subsequently, hydrogenation (by [H] atoms) of the intermediates, resulting in the formation of new compounds. At the completion of 8 h of degradation, the total production of the major product 4-methylanisole was 343.3 (g/kg lignin) with the following ideal conditions: aspen lignin concentration 40 g/L, current density 50 mA/cm^2^, and temperature 50 °C. Most of the aspen lignin was degraded after 8 h of electrolytic degradation. The electrocatalytic degradation of lignin is a useful strategy for converting lignin into useful compounds such as 4-methylanisole, which not only increases lignin’s potential value but also creates excellent chemicals instead of fossil raw materials.

Zhu and co-workers investigated a new green and efficient electrochemical approach for lignin depolymerization in which lignin in the alkaline electrolyte was directly electro-oxidized on the anode and chemically oxidized by the electro-generated H_2_O_2_ created on the cathode at the same time [32]. The linkages between C9 units in lignin (C–C and C–O–C linkages) were broken down, and more than 20 different LMW aromatic compounds that contain various functional groups, such as aldehydes, ketones, phenols, and acids, were generated and identified using GCMS and ESI-MS/MS measurements. It was discovered that O_2_ generated on the anode could be effectively converted to H_2_O_2_ in situ on the cathode. Following that, the electro-generated H_2_O_2_ in the alkaline electrolyte degraded further into a series of reactive oxygen species (ROS), enhancing lignin depolymerization. The effects of different electrolysis settings on H_2_O_2_ concentrations, H_2_O_2_ decomposition rates into reactive oxygen species (ROS), and LMW product yields were studied in depth. It was detected that by altering the current density and temperature, the concentration of electro-generated H_2_O_2_ and ROS could be controlled. It was observed that H_2_O_2_ and ROS play critical roles in lignin depolymerization. Larger yields of LMW products were predicted by the electrolysis settings for creating higher quantities of H_2_O_2_ and ROS. After electrolysis for 1 h at 80 °C at a current density of 8 mA cm^−2^ with an additional O_2_ supplement, 59.2% of lignin was depolymerized into LMW products, implying that the electric amount was reduced by 80% when compared to no extra O_2_ supplement. It was discovered that prompt separation of the products prevented further oxidation of the products, hence increasing the electrolytic yield. For the manufacturing of renewable chemicals, this method offered the complete exploitation of by-product O_2_, mild conditions, energy savings, greenness, and sustainability.

An electrocatalyst made of Co core/Pt partial shell nanoparticle alloy has also been used for the electrochemical oxidation of lignin to low molecular weight fragments and monomeric oxidation products [44]. More specifically, heptane and apocyanine were major chemical compounds that were identified. It was shown that these products were stable during the reaction and their concentration increased over time which showed that they did not further oxidize or decompose to lower fragments. However, a few other oxidation products, for example, 1,3-bis(1,1-dimethylethyl)benzene and 1,4-di-tert-butylphenol increased in the beginning and then decreased which showed further electrochemical reactions or decomposition mediated by the hydroxyl radicals. Over this alloyed catalyst, both homogeneous and heterogeneous electrochemical reactions competed with each other which makes the mechanistic investigations difficult. However, it was concluded that the electrochemical oxidation process is quasi-reversible and it involved heterogeneous charge transfer rate constants. It was concluded in this study that it is important to control the electrode potential so then specific products can be obtained more selectively. In summary, electrochemical oxidation offers several advantages but the main limitations of electrochemical oxidation are poor catalytic efficiency, radical formation, and high cost of metal electrodes.

### 3.2. Combined Oxidation and Electrocatalytic Hydrogenation Approach

Electrocatalytic hydrogenation (ECH) is a judicious combination of catalytic and electrochemical methods and it offers several advantages over catalytic hydrogenation and hydrogenolysis. ECH can be performed at ambient temperature and pressure, avoids the use of external hydrogen, has no catalyst poisoning, and offers greater mass transport due to in situ hydrogen production [45,46]. Moreover, selectivity can be controlled by controlling the cell potential [47]. A combined electrochemical oxidation and electrocatalytic hydrogenation (ECH) method for bamboo lignin at Cu cathode and Pb/PbO_2_ anode in alkaline media are reported by Liu and co-workers [48]. The reaction was optimized in terms of lignin concentration, current density, and temperature. They observed that a higher concentration of NaOH solution damaged the Pb/PbO_2_ electrode surface while a higher concentration of lignin (0–50 g) increased the potential toward more positive (−1.566 V to −1.378 V) for hydrogen evolution reaction. At the same time, an increase in reaction time promoted hydrogen evolution. The evolved hydrogen gas was chemisorbed at the surface of the cathode and participated in the hydrogenation reduction of intermediates. They observed that 1 mol L^−1^ of lignin, 20 mA/cm^2^ current density, 40 °C, and 2 h of electrolytic reaction time promoted the formation of monomeric products in higher yields. GCMS analysis of the product mixture showed 24 different products and among those *p*-coumaric acid (29.6 g/kg of lignin), vanillin (36.1 g/kg of lignin), and syringaldehyde (57.3 g/kg of lignin) were major products.

In a similar study by Cai and co-workers, corn stover lignin was degraded at a Cu/Ni-Mo-Co cathode and Pb/PbO_2_ anode in alkaline media via combined electrochemical oxidation and electrocatalytic hydrogenation mechanism [49]. They also observed that the oxidation efficiency of Pb/PbO_2_ anode was maximum at 1 M NaOH conc. and higher concentration and increased reaction time (>2 h) damaged the oxide layer on the surface of the electrode. Similarly, they also noticed more oxygen evolution with increasing lignin concentration from 10 to 40 g/L. Their optimized experimental conditions were like what Liu and co-workers reported earlier [48]. GCMS analysis revealed the formation of 24 different products with trans-ferulic acid (22.4 g/kg of lignin), vanillin (11.1 g/kg of lignin), 3-hydroxy-4-methoxyphenyl-ethanone (2.4 g/kg of lignin), syringaldehyde (10.0 g/kg of lignin), acetosyringone (6.9 g/kg of lignin), and 4-methoxy-3-methyl-phenol (38.3 g/kg of lignin).

In a combined electrocatalytic oxidation and electrocatalytic hydrogenation approach, Lan and co-workers reported degradation of cornstalk lignin at Pb/PbO_2_ anode and Ni cathode in an alkaline media [50]. An electrocatalytically produced ^.^OH radical and O_2_ anion from the Pb/PbO_2_ electrode were responsible for C–C and C–O bond cleavages. Hydrogen atoms generated at the Ni cathode by water reduction (Equation (4), Volmer reaction) were adsorbed on the surface of the cathode while lignin intermediates also adsorbed on the cathode surface (Equation (5)) thereafter adsorbed hydrogen H_ads_ hydrogenated lignin intermediates in electrochemical hydrogenation reaction (Equation (6)). The hydrogenated intermediates were desorbed from the cathode surface and in the meanwhile hydrogen evolved following the Tafel (Equation (7)) or hyrovsky reaction (Equation (8)). They also observed that hydrogenation reaction competed with the hydrogen evolution while low current densities favored hydrogenation. Moreover, temperature and lignin concentration also significantly affect monomer formation. According to their results, 35 °C and 40 g/L of lignin was found to be the optimal condition for monomer production. GCMS analysis revealed the formation of twelve different products with toluene, anisole, *o*-xylene, and *p*-xylene as major products with 36.1, 9.5, 14.4, and 11.7 g/kg of lignin respectively.

(4) $$M_{eled}+2e^{-}+2H_{2}O\;\;\;\;\;\;\;\;\;\to \;\;\;\;\;\;2H_{ads}\left(M_{eled}\right)+2OH^{-}\;\left(volmer\;reaction\right)$$

(5) $$M_{eled}+R\;\;\;\;\;\;\;\;\;\to \;\;\;\;\;R\left(M_{eled}\right)\;\;\;\;\;\;$$

(6) $$R\left(M_{eled}\right)+2H_{ads}\;\left(M_{eled}\right)\;\;\;\;\to \;\;\;\;\;RH_{2}\left(M_{eled}\right)\;\;\;\;\;\;\;\;\;\;\;\;$$

(7) $$H_{ads}\left(M_{eled}\right)+H_{ads}\left(M_{eled}\right)\;\;\;\to \;\;\;\;M_{eled}+H_{2}\;\;\;\;\;\;\;\;\;\;\;\;\;\left(Tafel\;reaction\right)$$

(8) $$H_{ads}\left(M_{eled}\right)+2\;e^{-}+2H_{2}O\;\;\;\to \;\;\;H_{2}+M_{eled}+OH^{-}\;\;\;\;\;\;\left(Heyrovsy\;reaction\right)\;$$

Anodic oxidation of rice-straw lignin at 35–55 °C in alkaline media using Ti/SnO_2_-Sb_2_O_3_/α-PbO_2_/β-PbO_2_ anodes and Ti/Fe, Ti/Cu, and Ti/Cu/Sn as cathodes is also reported where breakage of chemical bonds at the phenylpropyl linkage resulted in the formation of 16 different compounds [51]. First, oxidation of lignin took place at the anode followed by subsequent electro-reduction from the chemisorbed hydrogen which was generated at the metal cathodes. Six different products were generated from reduction following the same mechanism as explained above by Lan and co-workers [50] while the remaining ten products were obtained from the oxidation reaction following mechanism as below. They observed that oxidation of lignin took place from chemisorbed active oxygen formed on the anode surface therefore it is compulsory to overcome the reaction of Equation (12) (OER) in order to oxidize lignin. Aromatic ketones, aromatic aldehydes, and aromatic acids were observed in 4.09, 1.53, and 23.9 g/kg of lignin. The reaction was optimized for current density, reaction time, temperature, and lignin concentration through single-factor experiments and their results showed that reaction is controlled by current density (10–40 mA/cm^2^), time (5 h), and electrode material.

One limitation of ECH is the competitive reaction between hydrogenation and hydrogen evolution which can lower the overall faradaic efficiency of the process.

(9) $$M_{eled}Ox+e^{-}+OH^{-}\;\to \;\;\;\;M_{eled}Ox\;\left(\dot{O}H\right)\;\;\;\;\;\;\;\;$$

(10) $$M_{eled}Ox+e^{-}+OH^{-}\;\to \;\;\;\;M_{eled}Ox\;\left(\dot{O}H\right)$$

(11) $$M_{eled}Ox\;\left(\dot{O}H\right)+e^{-}+\;OH^{-}\;\;\to \;\;\;\;M_{eled}Ox^{+1}+H_{2}O\;\;\;\;\;$$

(12) $$M_{eled}Ox^{+1}\;\;\;\to \;\;\;\;\;M_{eled}Ox+\frac{1}{2}\;\;O_{2}\;\;\;\;$$

(13) $$\left(lignin\right)M_{eled}+M_{eled}Ox^{+1}\;\;\to \;\;\;\;M_{eled}Ox+RO\;\;\;\;\;\;\;$$

### 3.3. Integrated Approach

Several electrochemical methods are reported where an electrochemical step is integrated with another subsequent technical method in order to increase the process efficiency. A few of them are reported here. A combination of the photochemical and electrochemical method for oxidation of kraft lignin is reported by Chen and co-workers where Ta_2_O_5_-IrO_2_ was used as an electrocatalyst and TiO_2_ nanotube array was used as a photocatalyst [52]. In situ UV-Vis spectrometry was used to quantify lignin degradation. They noticed that lignin degradation efficiency significantly increased (92% degradation after 2 h) with photoelectrochemical oxidation in comparison to electrochemical oxidation (66% degradation after 2 h). Moreover, a combination of photoelectrochemical oxidation also increased the rate constants twice as higher as that of electrochemical oxidation. The rate constant for lignin oxidation was even higher than the combined rate constants obtained from both electrochemical and photochemical oxidation methods separately therefore the combination of both methods was more efficient. HPLC analysis showed the formation of vanillin and vanillic acid.

**Figure 1:**
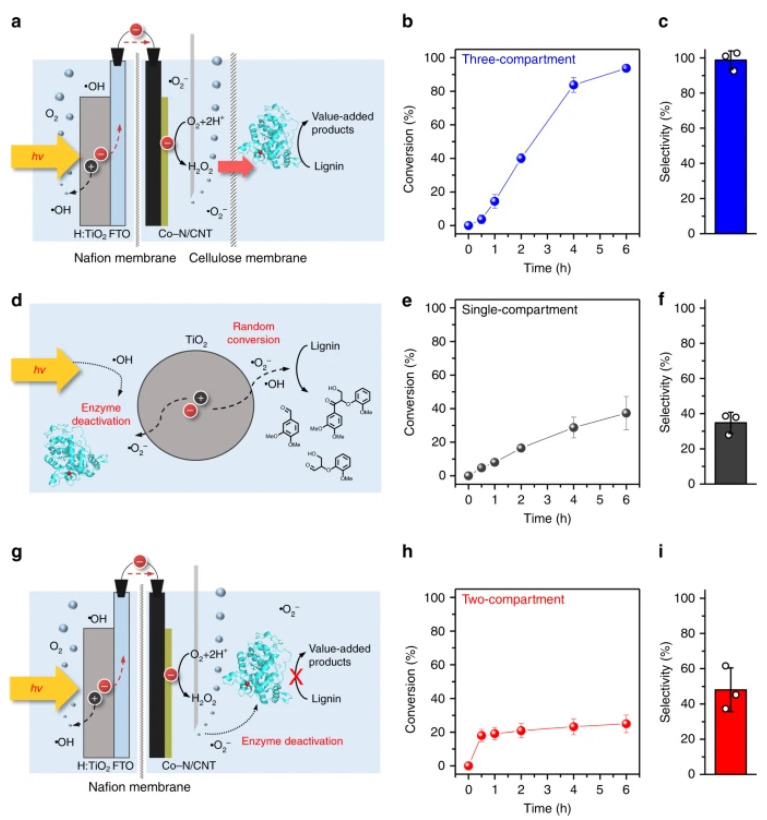
Schematic illustration of a photo-bio-electrochemical system for conversion of lignin model dimer in single (**d**), double (**g**), and three-compartment (**a**) systems. The (**b**,**e**,**h**) represent % age conversion vs time for coniferyl alcohol while (**c**,**f**,**i**) show formation of polymer yield after 6 h from three-compartment photo-electro-biochemical system (**b**,**c**), single-compartment powder-based photo-biochemical system (**e**,**f**), and two-compartment photo-electrochemicaal system (**h**,**i**), respectively. Standard deviation was used to show the error bars. Reprinted with permission from Ref. [54]. Copyright (2019) Springer Nature.

Lignin peroxidase has been used for selective cleavage of β-O-4 bonds in lignin but it requires an external source of H_2_O_2_ and its concentration mainly depends on enzyme stability [53]. The integration of electro(photo)chemical generation of H_2_O_2_ system with enzymatic catalysis is known to improve the process efficiency. Lignin peroxidase has also been used for depolymerization of β-O-4 lignin model compound and lignin in a three-compartment cell separated with Nafion and cellulose membranes together with TiO_2_ photocatalyst and Co-N/CNT as an electrocatalyst (Figure 1) [54]. This photoelectrobiochemical system enabled lignin dimer cleavage with high conversion efficiency (93.7%) and selectivity (98.7%) to 3,4-dimethylbenzaldehyde. Oxygen was reduced electrochemically at the Co-N/CNT cathode which generated the formation of hydrogen peroxide. The produced hydrogen peroxide entered the biocatalytic compartment and mediated the biocatalyst, lignin peroxidase, for depolymerization. Low selectivity and conversion efficiency was obtained in single and two-compartment cells. It was observed that a continuous low supply of hydrogen peroxide was compulsory for lignin peroxidase enzymatic activity.

In another study, an electrochemical membrane reactor was integrated with in situ nano-porous filtration units [55]. This reactor was made of a composite electrode mixer which contains a Ni rod electrode together with a 3D-printed static mixer. A tubular ceramic membrane consisting of aluminum oxide support with an active layer of titanium dioxide with a pore size of 1 nm was used inside the membrane reactor where anodic cleavage of kraft lignin took place and products were removed by applying slight overpressure. The combination of the electrode and 3D mixer also enhances the transport of product molecules. As a result, high molecular weight components (5434 Da) were retained while small molecules (342.8–426 Da) passed through the membrane and get separated. Moreover, fouling of the membrane was reported without the use of a static mixer.

### 3.4. Ionic Liquid/DES Assisted Approach

Very few studies are focused on the use of ionic liquids and deep eutectic solvents (DESs) as reaction media for electrocatalytic depolymerization of lignin. DESs have similar electrochemical properties as an ionic liquid [56], a broad operating electrochemical window, and additionally, avoid further oxidation and decomposition of the products. DESs are a mixture of two or more compounds that are homogeneous liquids at room temperature. The main advantage of DES is non-toxicity, biodegradability, ease to prepare, non-flammability, unreactive to water, and its ability to solubilize lignin [57]. Electrochemical depolymerization of kraft lignin in a deep eutectic solvent (DES) composed of ethylene glycol and choline chloride is reported by Marino and co-workers in a series of two studies. In a first study, they tried liquid–liquid extraction after electrolysis, and in the later study, they combined in situ emulsion-based extraction [58,59]. In a first study, they used 0.5 and 1 V, and DES with and without water was used. Significant depolymerization and decrease in molecular mass were observed in both cases together with the formation of vanillin and guaiacol as major products. In order to avoid liquid–liquid extraction, they used emulsion-based extraction in their later study. The emulsion was composed of DES, water as aqueous phase, and MIBK as an extractant while lignin also acted as an emulsifying agent. This DES emulsion was electro-oxidized in the presence of Ni or graphite electrodes in an undivided cell at 2.5 and 3.5 V for 1 h. The emulsion was stable and the graphite electrode was electrochemically stable under applied voltage range, however, the nickel electrode suffered from corrosion. Besides this, the nickel electrode promoted a higher degree of depolymerization while the graphite electrode showed some lignin hydrogel formation as a result of the reaction of lignin with ethylene glycol and no depolymerization were observed. The products were extracted by centrifugation which disrupted emulsion and extractant was isolated which contained two low molecular weight fractions and a solid cake. They concluded that better electrode materials need to be evaluated in order to be used for efficient depolymerization of lignin in DES.

In comparison, ionic liquids have been shown to dissolve biomass [60] and are considered to be green solvents. They are highly viscous, non-corrosive, and re-useable. Their electrochemical stability, wider electrochemical window, high conductivity, and excellent lignin solubility are the properties that make them promising candidates in comparison to aqueous electrolytes. However, very few studies are reported where electrocatalysis is carried out under ionic liquids because of their high cost, availability, and toxicity issues. A protic ionic liquid, triethylammonium methanesulfonate, has been shown to electrocatalytically oxidize 5 wt% alkali lignin solution to low molecular weight products in a 6% yield at a mixed ruthenium–vanadium–titanium oxide electrode at 1–1.5 V [61]. The oxidation reaction took place between the surface of the anode and the lignin molecule. Vanadium was proposed to be responsible for the electrocatalytic activity as it served as single-electron oxidant V(v) → V(iv). The ruthenium and titanium oxides did not show any catalytic activity. Furthermore, protic ionic liquid assisted in the proton transfer mechanism needed for C_α_–C_β_ bond cleavage. This protic ionic liquid was stable below 1.7 V and started to decompose at this voltage. It was also noted that higher voltages promoted the formation of low molecular weight compounds which were observed via HPLC and GCMS. Gauiacol, syringol, vanillin, and vanillic acid among several other products were identified.

**Figure 2:**
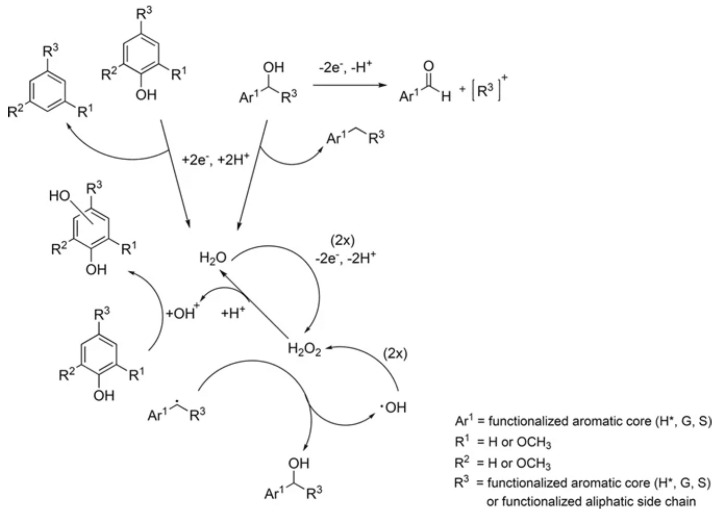
Schematic representation of electrochemical lignin degradation mediated by H_2_O_2_. The number of equivalent of raw materials required are given in the parenthesis. (H*), (G), and (S) represent 4-hydroxybenzyl, 3-methoxy-4-hydroxybenzyl, and 3,5-dimethoxy-4-hydroxybenzyl, respectively. Reprinted from permission from Ref. [62]. Copyright (2017) Springer Nature.

Later on, the same authors reported electrocatalytic degradation of two different kinds of lignin (alkali and organosolv) in an aprotic (1-ethyl-3-methylimidazolium trifluoromethanesulfonate; [emim][OTf]) and protic (triethylammonium methanesulfonate; TMS) ionic liquids with and without water at 2.5 V and 65 °C on vitreous carbon electrode [62]. The degradation of lignin was reported up to 23 and 90 wt% yields in both [emim][OTf] and [TMS] ionic liquids, respectively, which lead to the variety of different oligomeric and monomeric products identified by GCMS. The presence of water not only promoted the formation of hydrogen peroxide but also assisted in proton transport reactions which significantly influenced the efficiency of lignin degradation. This electrochemically produced hydrogen peroxide also promoted lignin degradation (Figure 2). It was observed that applied voltage and conc. of dissolved lignin had shown great impact on degradation efficiency of lignin.

In another study, Hempelmann and co-workers evaluated the performance of mixed metal oxide electrodes on the alkali lignin degradation in triethylammonium methanesulfonate [Et_3_NH][MeSO_3_] ionic liquid when electrocatalytic oxidation was carried out at 1.8 V for 3 h at 70 °C [63]. The ionic liquid used was regenerated up to 99% yield by aqueous extraction without any degradation which showed its electrochemical and thermal stability. Ternary mixed metal oxide, Ru_0.2_Mn_0.2_Ir_0.6_O_x_ has been shown to give the best results for degradation and the highest yield of low molecular weight products of up to 11.5 wt% identified via GCMS and direct infusion high-resolution mass spectrometry (DI-HRMS). It is reported that the catalytic activity of electrodes toward specific products can be obtained by the addition of certain metals in the electrode material. *p*-Coumaric acid, 4-hydroxy-3,5-dimethoxy cinnamaldehyde, 4-hydroxy-3,5-dimethoxy acetophenone, and vanillin were obtained from Ru_0.2_Mn_0.2_Ir_0.6_O_x_, Ru_0.2_Pd_0.2_Ir_0.6_O_x_, Ru_0.2_V_0.2_Ir_0.6_O_x_, and Ru0_.2_Ti_0.2_Ir_0.6_O_x_ electrodes, respectively.

## 4. Targeted/Product-Based Approach

These electrochemical methods focused on the selective production of commodities and useful chemicals which can be used as a building block. However, very few studies are reported so far regarding targeted electrochemical synthesis from lignin biopolymers. An overview of these methods toward specific products is discussed here.

### 4.1. Vanillin

Vanillin is mostly produced by oxidation with nitrobenzene in alkaline media up to 1–13 wt% yields [64,65,66]. However, vanillin obtained from this method cannot be utilized directly in food applications due to the toxic and carcinogenic nature of nitrobenzene. The vanillin gets contaminated by the formation of other nitrogenous waste by-products, for example, aniline and azobenzene. Vanillin is also known to be produced in 15 wt% yields by oxidative degradation in the presence of sodium hypochlorite and TEMPO nanocatalyst [67]. However, industrial production of vanillin involved copper-catalyzed aerobic degradation of lignosulfonate under elevated temperature and high oxygen pressure (15 bar) [68]. Due to the presence of copper, expensive purification methods are needed. The utilization of electricity as an oxidizer for the greener production of vanillin avoids the use of harmful and toxic metals as well as avoids expensive purification technology. Below we review some of the electrocatalytic methods for the synthesis of vanillin.

The electrochemical production of vanillin in double compartment batch and filter-press flow cell by oxidative degradation of kraft lignin is reported by Parpot and co-workers [69]. They used different metal electrodes, Ni, Cu, Au, Pt, PbO_2_, and DSA. The optimum yield was obtained at the highest applied current with the shortest reaction time. The electrochemical degradation was initiated by the oxygen produced at one of the electrodes and applied current density was the controlling parameter. A maximum conversion of 10% was obtained in the batch cell whereas, in the flow cell, 7–17% conversion was obtained. Methods for electrochemical degradation and selective production of vanillin and low molecular weight phenols from kraft lignin or directly from black liquor using nickel as an anode under high-temperature conditions (100–160 °C) were developed by Waldvogel and co-workers. Vanillin can be selectively adsorbed on basic anion exchange resins and regenerated by acidic treatment [70,71,72]. They reported that 2.7 C per mg of lignin and 10.0–12.5 mA/cm^2^ of current density gave the highest yields of vanillin. In another study, 5–7 wt% production of vanillin is reported in a filter-press cell at elevated temperature and pressure from anodic oxidation of lignosulfonates on a nickel-based electrode [73].

Zirbes and co-workers investigated the electrochemical oxidative degradation of lignin with various commercially available transition metal alloys (Ni, Co, Fe, and Ti-based materials) in alkaline media [74]. It has been found that vanillin and acetovanillin are the only low molecular products observed in the investigations in 0.6–0.7 wt% yields, respectively. Among all, Co and Ni-based materials have shown higher yields of vanillin as an oxidative product of lignin, but Ni-based materials proved better because of their corrosion stability. Overall, the selectivity of the degradation process applying Ni- and Co-based electrode materials is outstanding. The consecutive use of Ni foam electrodes for the electrochemical oxidative degradation of lignin led to a slight increase of the yield of vanillin from 0.6 wt% up to 0.9 wt%. It was observed that repeated use of Ni foam electrodes resulted in significantly increased yields of vanillin (160% increases), indicating a modification of the electrode surface. From the systematic investigation, it has been observed that increased activity was due to the formation of adsorption layer (catalytic active layer) at the electrode surface containing diamino-toluene as a major compound, which indicated that this compound is partly responsible for the activation process. It may be attributed that deposition of such an organic surface enhances the lipophilicity of the electrode surface, which increased the adsorption and oxidation of lignin, resulting in a higher yield of vanillin.

IrO_2_ is also reported to catalyze lignin oxidation to vanillin and vanillic acid [75]. Four different Ir-based electrodes, Ti/SnO_2_-IrO_2_, Ti/RuO_2_-IrO_2_, Ti/Ta_2_O_5_-IrO_2_, and Ti/TiO_2_-IrO_2_ were evaluated for their electrocatalytic activity and stability. The results showed that Ti/Ta_2_O_5_-IrO_2_ exhibited high activity for OER and low activity for lignin oxidation. Meanwhile, Ti/RuO_2_-IrO_2_ showed the highest stability, lifetime, and highest catalytic activity for lignin oxidation. They observed that the optimal time for vanillin and vanillic acid production is 45 min at 500 mA/cm^2^ is the maximum current density used for Ti/RuO_2_-IrO_2_ electrolytic activity. According to their in situ kinetic analysis via UV-Vis spectroscopy, they concluded that lignin oxidation followed pseudo-first-order kinetics on these electrodes. Moreover, the highest rate constant of 9.9 × 10^−3^ min^−1^ was observed for Ti/RuO_2_-IrO_2_ electrode for lignin degradation with an activation energy of 20 Kj/mol.

Similarly, in another study, a Ti/TiO_2_NT/PbO_2_ anode was used for the oxidation of kraft lignin and vanillin together with vanillic acid was observed as the main products via HPLC [76]. PbO_2_ nanoparticles were photochemically and electrochemically deposited on TiO_2_ support nanotubes and the results showed that TiO_2_ nanotubes increased the PbO_2_ geometric area, catalytic efficiency, and longer operational lifetime. The reaction kinetics were studied by UV-Vis spectrometry by correlating the absorbance at 290 nm with lignin concentration and the results showed that reaction followed pseudo-first-order kinetics with an activation energy of 16.0 kj/mol at 100 mA/cm^2^. It was found that lignin concentration has negligible effect while higher temperature increased the reaction rate and 100 mA/cm^2^ at 60 °C was found to be the optimal reaction condition for oxidation.

### 4.2. Phenolic Compounds

Chen and co-workers investigated the electrocatalytic method to decompose wheat straw lignin (WSL) through oxidation on Pb/PbO_2_ followed by subsequent electrocatalytic hydrogenation on a variety of alloyed materials (as cathodes) in alkaline solution [77]. The gel permeation chromatography (GPC) demonstrated that the average molecular weights (M_w_) of the lignin decreased in the electrocatalytic process using alloyed materials as cathodes and the resultant structures (derived from lignin) were electrocatalytically hydrogenated to guaiacyl (about 36–42 wt%), syringyl (25–33 wt%), and phenol-type (11–18 wt%) compounds. More specifically, the maximum yield of guaiacol, 4-ethylguaiacol, 4-propyl guaiacol, vanillin, acetovanillone, and vanillyl acetone were 1.134, 0.364, 1.040, 3.797, 1.248, and 1.147 g/kg lignin, respectively, and were obtained after 5 h of electrocatalytic decomposition. A variety of alloyed materials have been characterized and evaluated as electrocatalysts for the hydrogenation of wheat straw lignin (WSL) derivatives in a basic solution. Based on these findings, it was found that the three cathode materials, e.g., FeW_9_Cr_4_V_5_Co_3_, FeNi_8_Cr_18_ (304 alloyed steel), and FeW_9_Cr_4_Mo_3_V, had a greater effect on the enhancement of degradation products and electrocatalytic hydrogenation of lignin than other alloyed cathodes. Based on the total yield of guaiacyl-type compounds at the optimum conditions, 304 alloyed steel performed better than the other two preferred alloys in terms of lignin decomposition. Furthermore, these alloyed material offers the benefits of being cheap and anticorrosive. According to the proposed mechanism, the adsorbed hydrogen atom was generated in situ on the cathode surface by water electrolysis which is followed by an electrochemical hydrogen desorption step (Equation (14)), then the chemisorbed hydrogen reacted with the adsorbed lignin or intermediate compounds (Equations (15) and (16)), and finally, the reacted products desorbed from the surface of the electrode (Equation (17)). However, hydrogen gas (H_2_) evolution generated on the electrode (Equations (17) and (18)) surface was also observed as the side reaction.

(14) $$H_{2}O+M+e\;\;\;\;\to \;\;\;\;\;MH_{ads}+OH\;\;\;\left(Volmer\;reaction\right)\;$$

(15) $$R+M\;\;\to \;\;\;\;R_{ads}M$$

(16) $$2MH_{ads}+R_{ads}M\;\;\;\to \;\;\;\;RH_{2}M$$

(17) $$RH_{2}M\;\;\;\;\to \;\;\;\;RH_{2}+M$$

(18) $$\;MH_{ads}+H_{2}O+e\;\;\to \;\;\;\;M+H_{2}+OH\;\left(Heyrovsky\;reaction\right)$$

### 4.3. Hydrogen

Hydrogen is mostly produced on an industrial scale from steam reforming of natural gas, i.e., fossil fuels which require really high temperatures (>600 °C) [78]. The obtained hydrogen is less pure and highly contaminated with carbonaceous species (CO, CO_2_). This also requires additional purification steps. Hydrogen production has recently gained interest by electrolysis of water as it is the green method for hydrogen production [79]. The limitation is that water electrolysis is a very energy-intensive process as it requires a high amount of electrical energy which makes this process less economically feasible. Electrolysis of organic molecules (methanol and ethanol) is less energy demanding [80,81]. In this regard, there is a need to find new resources and solutions for the sustainable production of hydrogen. Utilizing lignin could be one alternative. Lignin electrolysis has also been used for the production of hydrogen gas in more than 90% faraday efficiency and with 40% less energy consumption in comparison to alkaline-water electrolysis [82]. In a proton-exchange membrane electrolyzer (PEM) polyoxometalate (POM) or ferric chloride (FeCl_3_) were used as charge transfer mediator and catalyst to directly oxidize lignin in 15% yield at carbon anode under mild reaction conditions (100 °C after 18 h) at a current density of 0.1 Acm^−2^. Several different kinds of lignin, kraft, alkali, and sulfonated lignins were used. Aromatic chemicals (vanillin, guaiacol, phenol, 1,2-dimethoxybenzene, and 3,4-dimethoxybenzaldehyde) were observed from oxidative lignin depolymerization by cleavage of most ether bonds (β-O-4, β-β, β-5) together with a reduction of hydroxyl groups in lignin structure. Oxidation of lignin occurred with simultaneous reduction of POM_red_ or Fe^+2^ while these reduced catalysts regenerated completely during the electrolysis reaction. According to the mechanism, first, oxidation of lignin by POM formed the H-POM_red_ complex and oxidized the products (Equation 19). This H-POM_red_ complex simultaneously generated electrons to the anode and protons to the electrolyte solution. The protons get diffused to the cathode side by PEM and finally get reduced to produce hydrogen gas. The authors concluded that simultaneous production of hydrogen gas together with high-value chemicals can increase process efficiency and reduce energy consumption.

(19) $$POM_{OX}OX+Lignin+H_{2}O\;\;\to \;\;\;\;H-POM_{red}+CO_{2}+oxidized\;products\;\;$$

(20) $$H-POM_{red}\;\;\;\;\to \;\;\;\;POM_{OX}Ox+e^{-}+H^{+}\;\;\;\;\;\;\left(at\;anode\right)$$

(21) $$e^{-}+H^{+}\;\;\to \;\;\;\frac{1}{2}H_{2}\;\;\;\;\;\;\;\;\;\;\;\;\;\;\;\;\;\left(at\;cathode\right)$$

**Figure 3:**
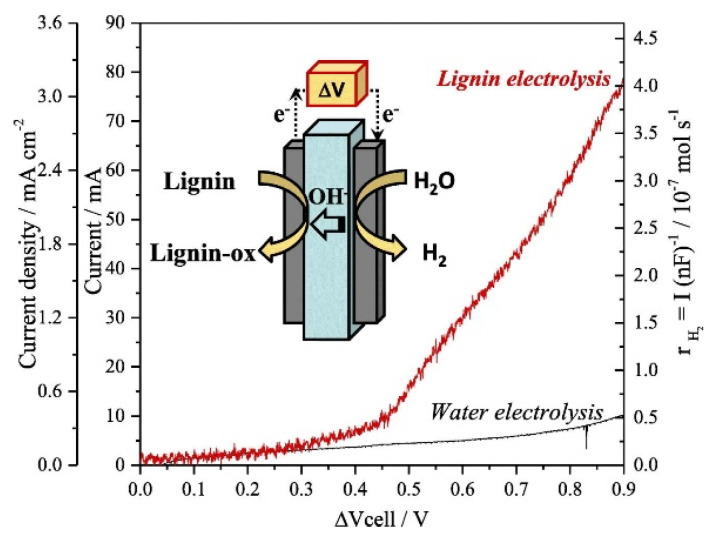
Schematic illustration of lignin electrolysis in polymer electrolyte membrane (PEM) electrolyzer in a continuous-flow system at 80 °C together with linear sweep voltammetry (0–0.9 V; sweep rate: 0.5 mV/s), anode: 10 g/L lignin (1 M NaOH), flow rate: 2.5 mL/min; cathode: NaOH (1 M), flow rate: 2.5 mL/min. Reprinted with permission from Ref. [83]. Copyright (2019) Elsevier.

In another study, a polymer electrolyte membrane (PEM) reactor was used for the sustainable production of pure hydrogen gas at approximately 0.45 V in a continuous flow from lignin electrolysis [83]. The electrochemical cell was composed of Pt-Ru/Fumapem (-OH conductor)//Pt/C representing anode//anion exchange membrane/cathode assembly (Figure 3). It was observed in the study that increases in temperature (30–90 °C) enhance the reaction efficiency for hydrogen production. In the absence of alkaline lignin solution, water electrolysis and production of hydrogen were not observed even at 80 °C at the potential range of 0 V – +0.9 V. In the presence of lignin, the production of hydrogen was observed at a 0.21 times lower potential than required normally for water electrolysis. Hydrogen was observed in 0.4 μmols^−1^ as a maximum under the studied reaction conditions. Moreover, oxygen evolution was not observed because lignin oxidation took place at voltages lower than required for OER reaction. However, no oxidation products of lignin was observed at the anode.

### 4.4. BHT

Degradation of lignin on Pb/PbO_2_ anode in alkaline solution at 25 mA/cm^2^ after 3 h of anode electrochemical catalysis is reported to generate 2,6-bis(1,1,-dimethylethyl)-4-methyl phenol (BHT) in a 7.0% yield [84]. Acetic acid and 4-hydroxy-3-methoxybenzaldehyde were also observed via GCMS. BHT was not only separated and purified by column chromatography using petroleum ether but also identified using a number of analytical techniques, e.g., ^1^H NMR, ^13^C NMR, and GCMS. Furthermore, chemical stability testing of the electrode showed its decomposition after 4 h of electrolysis in 0.5 M NaOH. According to the proposed mechanism, superoxide anion radical (O^2−.^) was suggested to selectively oxidize the C–C bond in the aromatic ring and cleave sidechain hydroxylphenyl propane from the benzene ring. H^+^ produced on the cathode surface from water reduction then reduced the intermediate product and generate isobutylene carbenium ion. Simultaneously, lignin degraded to 4-methyl-phenol gets attacked by isobutylene carbenium ion in an electrophilic reaction and leads to the formation of BHT.

### 4.5. 1,3-diethylbenzene

1,3-diethylbenzene is an important intermediate used for the production of styrene and polystyrene. It is mainly generated from fossil-based chemistries i.e., alkylation of ethylbenzene in the presence of ethanol on HZSM-5 zeolite [85]. Catalytic fast pyrolysis of cordgrass lignin at 500 °C has also been used for the production of 1,3-diethylbenzene (0.1 wt% yield) [86]. However, these processes involve high temperature and pressure. Shen and co-workers reported that electrocatalytic hydrocracking of bamboo lignin in alkali can selectively produce 1,3-diethyl benzene in 0.361 g/g lignin from 30 g/L of lignin concentration [87]. A fixed bed 3D-electrode reactor was used in which Ti/SnO_2_-Sb_2_O_3_/α-PbO_2_/β-PbO_2_ was employed as an anode and the copper mesh was used as a cathode. Ceramic material was placed in between the cathode and anode. Complete decomposition of bamboo lignin was observed after 10 h of electrolysis at 25 °C at a current density of 25 mA/cm^2^ when 50 g/L of lignin concentration was used in 0.5 M NaOH. Together with 1,3-diethylbenzene, 46 different kinds of compounds were identified while only a few of them were successfully isolated by distillation under reduced pressure. It was proposed that the electrocatalytic hydrocracking followed two reaction sequences, electrocatalytic oxidation (ECO) followed by electrocatalytic hydrogenation (ECH). In the ECO process, anodic oxidation proceeds by cleavage of C_α_–C_β_ bond leading to cracked intermediates and oxidation products. Later in the ECH process, the C–O bond hydrocracked and produced H_ads_ atoms hydrogenate the intermediate products. Therefore, the production of 1,3-diethyl benzene also depends on the efficient absorption of hydrogen and the evolution of hydrogen.

### 4.6. Carboxylic Acid Production

Production of carboxylic acids from lignin is reported via a two-stage gas-phase catalytic process or by partial wet oxidation of alkali lignin [88,89]. Production of adipic acid involves the nitric acid process while this technology suffers from corrosion and formation of highly toxic NO_x_ species [90]. In comparison to these methods, electrochemical production of carboxylic acids from lignin offers a green and inexpensive approach. A simple homemade swiss-roll type of electrochemical reactor with nickel foam electrode was employed for the successful production of carboxylic acid (40 wt%) after electrocatalytic valorization of kraft lignin [91]. No complex reactor or expensive catalysts were needed to obtain value-added products. Reactions were performed at 0.8, 2.5, and 3.0 V for 0–420 min. The relative molecular weight of lignin was reduced to 50% (2.5 V) and 80% (3.5 V for 7 h) after depolymerization. A significant amount of oxalic acid (6.4%), formic acid (26.8%), and acetic acid (4.2%) while a low yield of vanillin was observed. A maximum 1.25 wt% yield of vanillin was obtained after 5 h at 0.8 V and further control experiments showed degradation of vanillin under electrochemical process which was responsible for its low production. It was found that oxalic acid and acetic acid were stable products whereas formic acid decomposed further during depolymerization reaction. Analysis of TOC showed low carbon loss. It was assumed that the depolymerization process was preceded by hydroxyl radicals.

Lignin-derived dicarboxylic acids have been produced over nickel foam electrode (NiOOH) by batch and flow electrolysis under alkaline media [92]. Alkylated cyclohexanols derived from lignin were electrocatalytically converted to five different alkylated dicarboxylic acids (adipic acid as major) in 64% total yield in a completely green and sustainable approach. A commercial flow cell was used for scaling up the procedure and no additional additive was required besides caustic soda in their system. 4-methylcyclohexanol was used as a lignin model compound for conversion to 3-methyladipic acid. The undivided cell was used in a galvanic setup for batch electrolysis experiments. Shorter chain carboxylic acids, such as succinic and glutaric acid, were also observed together with adipic acid. It was found that electrode morphology, such as the greater surface area in foam electrodes, increased the catalytic efficiency of the process. Dicarboxylic acid products were not observed when molybdenum, vanadium, and cobalt electrodes were used as anodes. Furthermore, lower current density (2.5 mA/cm^2^) at the larger surface area of the electrode gave the best results. It was found that the increase in temperature to 50 °C decreased the yield of the product because starting material is volatile. Moreover, a slightly higher charge (8.5 F) than the theoretically predicted one (8 F) promoted the reaction efficiently. Since only 20–50% yield could be obtained in the batch mode, a flow system was also optimized for higher conversion to the 3-methyladipic acid. According to the optimized results, a 64% yield of 3-methyladipic acid was obtained when using a flow rate of 12.5 mL/min via membrane pump over the anodic surface area of 20 mm × 60 mm × 6 mm nickel foam.

## 5. Electrolytic Upgrading of Bio-Oil/Lignin Model Compounds

**Figure 4:**
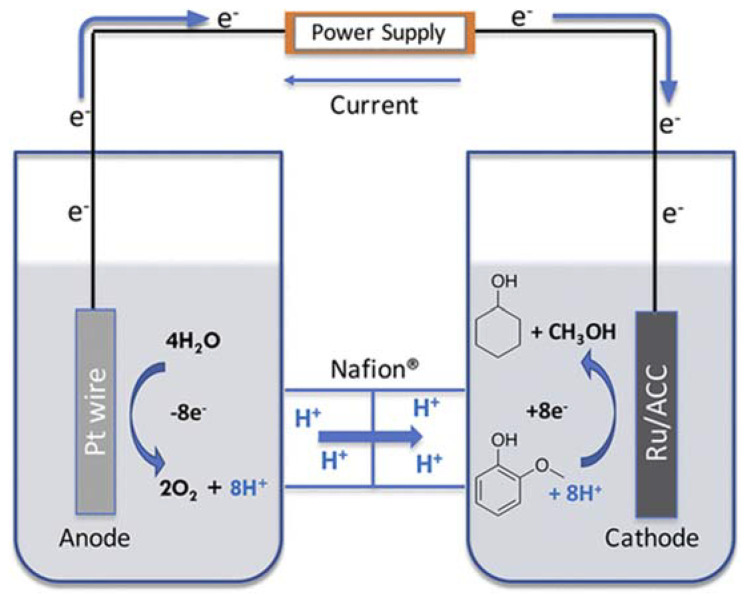
Schematic illustration of divided cell separated by Nafion-117 membrane. Reprinted with permissions from Ref. [96]. Copyright (2019) American Chemical Society.

Upgrading of lignin-derived model compounds and bio-oil is extensively studied by catalytic hydroprocessing but it suffers from high capital and operating costs [93]. In contrast, electrocatalytic hydrogenation (ECH) offers mild conditions and avoids mass transfer and hydrogen gas solubility problems [94]. The low temperature used in ECH avoids catalyst poisoning which potentially reduced the process cost. Integrating pyrolysis with electrocatalysis for the production of fine chemicals and fuel could significantly increase the process efficiency and profitability of biorefineries. Fast pyrolysis of lignin to deconstruct the lignin structure and obtain bio-oil first, followed by subsequent electrocatalytic hydrogenation to obtain deoxygenated and saturated products, could be a promising strategy. Only a few examples are discussed for a short overview of the technology. A review is recommended for a detailed overview [95]. Ru/ACC was used as a cathode at mild temperature (80 °C) for ECH of twenty different model compounds including phenol, guaiacol, syringol, alkyl phenol, aryl-methyl ethers, and several others which are mainly present in the lignin bio-oil [96]. The ECH reaction was carried out in a divided cell separated by Nafion 117 membrane, 0.2 M HCl solution and 0.2 M phosphate buffer were used as catholyte and anolyte (Figure 4). Hydrogenation together with C–O and C–C bond cleavage was observed. An increase in alkyl chain length not only decreased the conversion efficiency while selectivity for demethoxylation was also reduced. Conversion of guaiacol was increased in the presence of vicinal hydroxyl or methoxyl groups. On the other hand, faradaic efficiency was increased with the increased initial concentration of substrate.

Effect of electrolyte and electrocatalyst on the electroreductive hydrogenation–hydrogenolysis (ECH) of lignin model compounds (phenol and gauiacol) was investigated in a stirred slurry electrochemical divided reactor separated by Nafion 117 membrane under mild reaction conditions (50 °C, 1 atm, 4 h) [97]. Acid (H_2_SO_4_)–acid and acid–neutral (NaCl) electrolyte pairs were used as catholyte–anolyte pairs in the presence of three different electrode systems (Pt/C, Ru/C, and Pd/C) at a current density of 109 mA/cm^2^. Pt/C showed higher activity in acid–acid electrolyte pair (pH < 0.8) due to higher surface area and metal dispersion while neutral–acid electrolyte pair performed best as catholyte-anolyte pair when Ru/C or Pd/C was used. Overall, Ru/C showed the highest catalytic activity when pH was 9–11. The order of catalytic activity which was observed for guaiacol was Pt/C/Ru/C/Pd/C. 2-methoxycyclohexanol was obtained as the major product from the ECH of guaiacol. However, product selectivity changed significantly when the catholyte–anolyte pair was NaCl (0.2 or 0.5 M)-H_2_SO_4_ (0.2 M) thereby resulting in the formation of cyclohexanol and cyclohexanone at 50 °C on Pt/C electrode. For ECH of phenol, it was reported that catalytic activity decreased in the order of Pt/C > Pd/C > Ru/C irrespective of electrolyte pairs used. Higher faradaic efficiencies and conversions of up to 96 and 100%, respectively, were obtained.

In another study, similar stirred slurry electrochemical reactors as above were used for ECH of guaiacol in methanesulfonic acid (0.2 M) as an electrolyte over Pt/C electrode under mild reaction conditions (1 atm, 30–60 °C) [98]. It was found that stirring rate is critical since it affects mass transfer reaction between substrate and electrode surface and helped in maintaining uniform charge distribution throughout the reaction chamber. An optimal stirring of 350 rpm resulted in 100% guaiacol conversion with 78% faradaic efficiency after 4 h at 1 atm and 40 °C when the reaction was carried out at a current density of 165 mA/cm^2^. It was observed that 5 wt% of Pt content increased both guaiacol conversion and faradaic efficiency in comparison to 1 wt% or 10 wt%. Guaiacol conversion was also dependent on the initial concentration, e.g., 48 to 100% conversion achieved when 4.8 to 13.1 wt% guaiacol was used, respectively. Higher temperatures (60 °C) promoted demethoxylation-ring saturation. ECH of guaiacol was observed to follow first-order or second-order kinetics according to the Langmuir–Hinshelwood model which is highly dependent on the initial guaiacol concentration and catalyst loading. It was also observed that the fastest step was the hydrogenation of phenol to cyclohexanol while demethoxylation of guaiacol was the slowest step.

## 6. Electrocatalytic Cleavage of Model Compounds

Similar to the above described electrochemical methods, electrocatalytic cleavage of the lignin model offers several advantages being green, inexpensive, and mild operating conditions (ambient temperature and pressure). The electrolysis of lignin using a polymer electrolyte membrane (PEM) alkaline electrolyzer with Pt-Ru-based anodes was previously described by Beliaeva and co-workers. The performance of Pt-Ru-based anodes was demonstrated over a wide range of applied potentials (1.2 V) and reaction temperatures (90 °C), although the system’s activity was severely hampered, resulting in poor H_2_ generation rates [99]. The goal of their investigation was to find out the major drawbacks of Pt-Ru electrodes for lignin electro-oxidation. The 2-phenoxyethanol (2-PE) was chosen as a model molecule for the β-O-4 linkage, which accounts for over 60% of the lignin biopolymer. When electrolyzed, phenoxyethanol (2-PE) exhibited a similar electro-oxidation pattern to that of simpler alcohols such as methanol, ethanol, and glycerol. Analyses of the anodic electro-oxidation products revealed that the primary product was phenoxyacetic acid (PAA). Furthermore, thermodynamic DFT studies showed an electro-oxidation process in which the 2-PE model molecule was converted to aldehyde before being further oxidized to carboxylic acid (PAA). The experimental set-up revealed that the performance of the electrolysis system degraded significantly over time, owing to the unlikelihood of depolymerization of lignin under the studied conditions (C–C cleavage is strongly suppressed), as well as the adsorption of reaction intermediates and products, resulting in a strong deactivation of the anodic catalyst.

Reductive electrocatalytic cleavage in aryl–aryl ether (C–O) linkages and β-O-4, α-O-4, 4-O-5 lignin model dimers mediated by NaBH_4_ and TBABH_4_ is reported to produce phenol and aromatics under air at room temperature mild reaction conditions [100]. A simple set-up with a single cell and Pt as the working electrode in 0.2 M TBABF_4_ (NaBF_4_) solution in DMF as an electrolyte was used. The electrocatalytic reduction was performed at 25 mA for 105 min. The reaction was optimized in terms of electrode material, mediator, and solvent. Their results showed that the RVC electrode can also be used in place of Pt as similar selectivity, catalytic activity, and conversions were observed when RVC was used as the cathode. In contrast, graphite electrodes as an anode gave slightly poor yields. Moreover, the working area of electrodes affects selectivity as Pt. foil showed better results in comparison to Pt (g). Acetonitrile, THF, and 1,4-dioxane gave poor yields while NMP gave the best results (95% yield). Finally, very low conversions were observed when a base other than NaBH_4_ was used. Moreover, no hydrogenation products, cyclohexene, cyclohexane, and cyclohexanol were observed which confirmed no evolution of hydrogen gas during the reaction conditions. Furthermore, it was proposed that the reaction proceed via one-electron reduction, and diaryl ethers get reduced on the cathode surface to form diaryl ether radical anion which then undergoes cleavage to phenyl radical and phenoxide anion. Phenyl radical reacts with hydrogen which is mainly generated from the solvent to form aromatics.

The effect of reactor type (single-cell vs. divided cell) on the electrocatalytic reductive cleavage of β-O-4 lignin model compound, 2-phenoxyacetophenone, was evaluated in the presence of DES (ethylene glycol and choline chloride in a 2:1 ratio with 10% water) at a Cu electrode [101]. Different monomers and oligomers were obtained depending on the type of reactor used, a higher number of carbonyl compounds was observed in the divided cell while hydroxyl-end products were obtained in a single cell. Interestingly, visible color differences were observed in both types of reactors, a nearly colorless solution was obtained after 20 h in a single cell in contrast to a darker colored solution when the divided cell was used which correlates to the higher amount of alcohols in single cells vs. ketones and ethers in the divided cell and this is also in accordance to the GCMS results. The authors concluded that a single cell promoted the oxygen evolution reaction (OER) which enhances ether cleavage leading to hydroxyl-end products. The presence of phenolic groups was observed in both reactor types according to NMR. However, products were also obtained that carry aliphatic side end groups originating from the side reaction between ethylene glycol and 2-phenoxyacetophenone. In general, product selectivity can be tuned by the proper design of the reactor.

Pardini and co-workers described the anodic oxidative cleavage of lignin model compounds at a nickel anode in alkaline media with and without external mediators [102]. They observed cleavage of model compounds to the corresponding aldehydes and carboxylic acid up to 40% yield together with polymerization. They concluded that the presence of β-O-aryl ether functionality, the 4-hydroxy group or the β-CH_2_OH group, or a carbonyl group at C_α_ is compulsory for cleavage. High temperature mediated chemical reaction was important before electrolysis is carried out. Moreover, the reaction is initiated by either single electron transfer or hydrogen atom abstraction at the electrode surface. NHPI/PINO mediated electrochemical oxidation of non-phenolic lignin model substrates showed that NHPI is an excellent mediator for selective C_α_-carbonylation of non-phenolic β-O-4 lignin model compounds. C_α_-carbonylation of dimeric lignin models was obtained in high yields (88–92 wt%) with NHPI when 2,6-lutidine was used as a base. Comparison of NHPI/PINO mediated reaction with that of the direct electrochemical oxidation of lignin models showed poor selectivity and yield toward oxidation (5–40%) which showed the potential of PINO as an excellent reagent [103]. TEMPO-mediated electrochemical oxidation of lignin monomer, anisyl alcohol, and a series of other alcohols was reported earlier and results showed the formation of anisyl aldehyde in 83% yield when 5 mol% TEMPO was used [104]. Nakatsubo and co-workers reported direct anodic oxidation of non-phenolic lignin model compounds, both dimeric β-O-4 models and simpler monomeric models, with(out) 2,6-lutidine as a base when carbon felt was used as an anode. Different electrochemical behavior was observed for both lignin models. Anodic oxidation of the β-O-4 model compound when promoted by base leads to the formation of benzyldehyde and phenol as products due to C_α_–C_β_ bond cleavage, whereas C_α_ carbonyl compounds in 60–80% yield are reported from the simpler monomeric lignin model (4-ethoxy-3-methoxyphenyl)ethanol. Without a base, the reaction did not proceed [105].

Similarly, another study is reported where oxidation of dimeric β-O-4 model was carried out in the presence of TEMPO. The results showed a strong influence of electrolytes on the chemoselectivity between C_α_-carbonylation and C_γ_-carboxylation. C_α_-carbonylation (2–11%) was observed when the LiClO_4_/MeCN-H_2_O system was used, whereas C_γ_-carboxylation in 72–93% yield was noticed when dioxane/phosphate buffer (pH: 7) was used. Moreover, they also noticed that the electrocatalytic efficiency of 4-acetamido-TEMPO was higher than the TEMPO alone [106].

One of the lignin model compounds, veratryl alcohol, can be oxidized to the corresponding aldehyde upon electro-enzymatic oxidation when lignin peroxidase was used as an active redox catalyst. This electro-enzymatic system used only the electrochemical reactor and the reaction can be performed without external hydrogen peroxide [107]. Hydrogen peroxide is one of the crucial oxidants needed for the enzymatic activity of the lignin peroxidase but when this enzymatic oxidation is coupled with electricity it does not require an external oxidant. In the electrolytic system, hydrogen peroxide was generated in situ when oxygen gets reduced in the presence of an acid, this in situ generated hydrogen peroxide then mediated the Lip enzyme. Once Lip activated, it oxidized veratryl alcohol. Lip showed a reasonable reaction rate even without the external addition of hydrogen peroxide. A cation exchange membrane was used to separate the anode from the rest of the reaction media. The anode was also used for proton generation by water oxidation at the same time.

In another study, electrochemical oxidation of veratryl alcohol is reported when a variety of manganese(III) complexes with Schiff base ligands Mn_2_L_2_(H_2_O)_2_(N(CN)_2_)_2_ were used as electrocatalysts [108]. The effect of different parameters such as temperature, reaction pH, concentration, redox activity, turnover number of catalyst, etc., on the reaction kinetics was evaluated. The highest catalyst activity was achieved at pH 8.25, at 40 °C, and a turnover number of 84 was achieved when 0.25% of catalyst was used. However, the redox potentials of different catalysts do not affect the catalytic efficiency and reaction rate.

**Figure 5:**
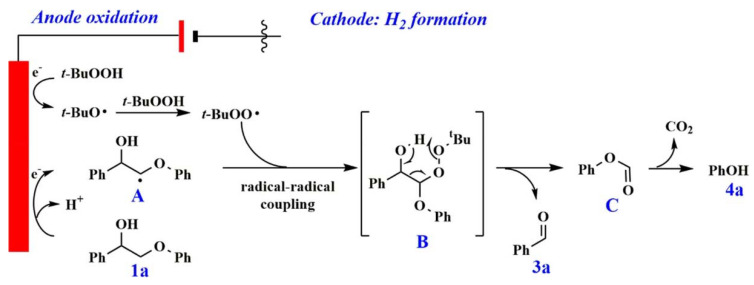
Mechanism of C_α_–C_β_ bond cleavage in lignin model compounds. Reprinted with permission from Ref. [109]. Copyright (2021) American Chemical Society.

A process for selective cleavage of C_α_–C_β_ bond in β-O-4 linkage in lignin model compounds in the presence of tert-butyl hydroperoxide in water as an oxidant is reported to produce high yields of aldehydes and phenol [109]. The electrocatalytic reaction took place under mild reaction conditions at room temperature in an undivided cell with a Pt as an anode while simultaneous production of H_2_ at the Pt cathode is also observed. Aldehydes were obtained in 67% yield with a faradaic efficiency of up to 12% at an electric current of 20 mA for 3 h. A decrease in yield is observed at lower current while increasing current further to 50 mA showed a positive increase in the yield. It was observed that an increase in the methoxy substituents decreased the reaction efficiency due to over-oxidation or further polymerization of the intermediates. It was shown that reaction occurred through the formation of the C_β_ radical initiated by the peroxide intermediate (Figure 5). The reaction strategy is shown to be applicable to real lignin.

Mehdavi and co-workers reported electrocatalytic hydrogenolysis (ECH) of lignin model compounds (benzyl phenyl ether, β-phenoxyethylbenzene, and α-phenoxyacetophenone) at a Raney Ni electrode in aqueous ethanol [110]. According to their results, the efficiency of the C–O bond hydrogenolysis was greatly influenced by substrate concentration, current density, and temperature. A decrease in current density from 160 to 20 mA/cm^2^ was shown to increase conversion and current efficiency and possibly due to decreased hydrogen evolution reaction. A direct relationship between substrate concentration and electrocatalytic efficiency was observed. Higher substrate concentrations led to an increased amount of ECH product, however, selectivity remained unaffected. The conversion was increased from 50 to 70% as a result of an increase in temperature from 25 to 40 °C. The effect of substituents, alkyl instead of phenyl, and hydrogen in place of methoxy groups were also investigated. It was found that steric interaction decreased the efficiency of the ECH process due to the poor adsorption of the substrate over the electrode surface. Their results showed that ECH is selective and an efficiency of up to 100% can be obtained by optimal choice of reaction parameters. Only 8% of hydrogenated and demethoxylated products cyclohexanol and phenol were also observed.

Cui and co-workers presented a new type of electrocatalyst for selective C_α_−C_β_ bond oxidative cleavage in lignin, electrocatalyzed by atomically dispersed Pt−N_3_C_1_ sites planted on nitrogen-doped carbon nanotubes (Pt_1_/N-CNTs), created via a stepwise polymerization−carbonization−electrostatic adsorption strategy [111]. Synthesized catalysts were shown to be highly active and selective toward C_α_−C_β_ bond cleavage in β-O-4 model compounds under ambient conditions. When compared to previously reported electrocatalysts, Pt_1_/N-CNTs achieved 99% substrate conversion with an 81% yield of benzaldehyde. Furthermore, Pt_1_/N-CNTs with only 0.41 wt% Pt produced much more benzaldehyde than the state-of-the-art bulk Pt electrode (100 wt% Pt) and commercial Pt/C catalyst (20 wt% Pt). The experimental analysis combined with DFT calculation reveals that the reaction occurs via a crucial C_β_ radical intermediate, which enables the particular C_α_−C_β_ bond breakage during the subsequent radical/radical cross-coupling phase. According to the proposed mechanism, first, the tert-butoxyl radical (tBuO·) and tertbutylperoxyl radical (tBuOO·) were generated from TBHP. Subsequently, the C_β_ radical was produced via C_β_−H abstraction of lignin model compound on the Pt_1_/N-CNTs N-CNTs anode surface. Lastly, tBuO·/tBuOO· reacted with the unstable C_β_ radical to generate intermediate C via a radical/radical cross-coupling reaction. The reaction was followed by electron transferring in C, inducing the cleavage of the C_α_−C_β_ bond and the generation of aromatic aldehyde, phenol, and CO_2_. H_2_ was simultaneously produced at the cathode via H_2_O reduction.

Fang and co-workers presented a thio-assisted electrocatalytic reductive approach using inexpensive reticulated vitreous carbon (RVC) as the working cathode to cleave the β-O-4-type linkages in keto aryl ethers [112]. In the presence of a pre-electrolyzed disulfide (2,2′-dithiodiethanol) and a radical inhibitor (BHT) at room temperature at a current density of 2.5 mA cm^−2^, cathodic reduction of β-O-4 models was afforded over 90% of the corresponding monomeric C–O cleavage into phenolic and keto monomers (phenol and acetophenone) with over 90% yields in only 1.5 h. The electrochemical method showed promising levels of cleavage and solubilization when applied to pre-oxidized lignin, owing to the use of thiols (serve as small diffusible redox carriers via the thiol/disulfide couple), enabling the decomposition of lignin into small cleaved 2-phenoxyacetophenone. According to the proposed mechanism, the reaction proceeds via the pre-reduction of the disulfide-formed active species which promoted cleavage of 2-phenoxyacetophenone. However, it was observed that for complete substrate conversion, an electric current must be supplied throughout the reaction time. Free thiyl radicals were not observed during dimer cleavage. The reaction proceeds faster under the N_2_ atmosphere than under air.

C–O bond cleavage in lignin model compounds is reported to proceed via anodic oxidation on the carbon electrodes at +1.1 V under room temperature conditions [113]. After 20 h, diaryl ethers completely degrade to C–O bond cleavage products. Anodic oxidation proceeds via direct electron transfer between the electrode and diaryl ethers. Surface modification was found to further accelerate the catalytic efficiency of the process.

*p*-Benzyloxyl phenol (PBP) one of the lignin model compounds has been degraded electrochemically in the protic ionic liquid ([HNEt_3_][HSO_4_]) in a non-membrane cell in the presence of oxygen reductive reaction (ORR) cathode [114]. It was observed that two-electron reductive products, H_2_O_2_, ^.^OH, ^.^O^2-^, and HO_2_ firstly formed from O_2_ were mainly reactive oxygen species (ROS) which promoted electrochemical degradation. The rate of degradation of lignin (48.2%) and current efficiency (29.5%) obtained in protic ionic liquids were higher than the ones obtained in the aprotic ionic liquids ([BMIM][BF_4_]). C–O bond cleavage products (benzyl alcohol, benzaldehyde, and benzoquinone) were identified by GCMS and it was observed that the protons available from the protic ionic liquid were also crucial for the degradation of the lignin model compound, and this was further confirmed by adding an amount of the external water to the protic liquid. Significant improvement in the efficiency of the process was observed which supports the role of proton in reaction efficiency. It was also identified that amount and lifetime of ROS improved the current efficiency and degradation process. According to the proposed mechanism, it was suggested that first PBP dissociates and released a proton/molecule, and converts to phenolate ions (PBP^−^). This anion then diffused on the surface of the electrode and formed phenoxy radical (PBP) after donating one electron/molecule. Some of the PBP radicals undergo a reversible reaction to form PBP anion again while other PBP radicals reacted with ROS species and underwent bond cleavage to benzyl alcohol and benzoquinone. Benzaldehyde was also observed as an oxidation product that originated from the reaction of benzyl alcohol and ROS.

Electrocatalytic hydrogenation (ECH) of 4-O-5 linkage in the lignin model compound has been reported on the ruthenium supported on activated carbon cloth as an electrode material in alkaline, acidic, and neutral media [115]. A range of lignin models containing 4-O-5 linkage, for example, 3-phenoxyphenol (3-PP), 4-phenoxyphenol (4-PP), 3-phenoxyanisole (3-PA), and 3-phenoxytoluene (3-PT) were investigated. Cyclohexanol (100% conversion) was observed from the 3-PP and 4-PP after C–O bond cleavage and further hydrogenation but 3-PA and 3-PT gave lower yields and conversions to cyclohexanol due to limited solubility. It was observed that the presence of oxygenated functional groups close to the ether linkage further promotes the C–O cleavage due to the electron-withdrawing effect. A higher yield was observed in the presence of an alkaline electrolyte. Faradaic efficiency was modestly increased to 25% when the concentration of substrate increased from 10 mM to 40 mM while a significant improvement in faradaic efficiency (up 96%) was observed when current density decreased from 33.3 mA/cm^2^ to 6.67 mA/cm^2^ which is possibly due to lower hydrogen evolution at less current density.

### Summary and Outlook

In this review, we provide a comprehensive summary and insights into the nature of electrocatalytic depolymerization of lignin together with lignin model compounds for the production of high-value chemicals. Electrocatalytic depolymerization of lignin driven by renewable electricity under mild reaction conditions, ambient pressure, and relatively lower temperatures give us benefits to tune the selectivity toward certain specific products. However, these processes are at a low technology readiness level and the techno-economic of these processes are not evaluated extensively so far. Nasarabadi and co-workers evaluated the cost of integration of an electrochemical reactor in a biorefinery setup [116]. They found out that if the rate of lignin conversion is increased then break-even product stream value may fall to 1–2 USD/kg. However, if the lignin conversion rate is low then the break-even product stream will be too expensive to be economically feasible.

Moreover, current technological processes suffer from several limitations and are often challenging. Often, a complex experimental setup is required and in some cases, high-temperature electrochemical batch reactors have been employed to increase product selectivity and conversion. Besides this, the evolution of hydrogen (HER) and oxygen (OER) are competing side reactions and proper voltages and electrode materials are required to decrease HER and OER reactions. Similarly, corrosion and fouling of electrode materials (Cu and other heterogeneous electrodes) sometimes occur in highly alkaline media due to the formation of an electrode–electrolyte interface on the surface of the electrode which hinders the applications of those electrodes to be used in certain processes. Sometimes, over-oxidation of products to carbon dioxide can take place without proper control of reaction parameters.

In situ product separations integrated with an electrocatalytic process could avoid further decomposition of products, for example, integration of membrane separation technology. Resins have been used in certain electrocatalytic processes for in situ product absorption but these could increase the price of the process and could be challenging to be employed when scaling up the technology. Depolymerization of radical cations or anions can be another limiting factor but this can be easily compensated in an electrochemical reaction by using relatively low concentrations of lignin.

Furthermore, the low faradaic efficiency of the process and the high cost of the electrode materials hampered the wide application of this technique. For downstream valorization of lignin to high-value chemicals, higher faradaic efficiencies and current densities are needed to make this technology profitable. Engineering and designing better electrode materials can be challenging and cumbersome. A better electrode design in terms of stability, loading, lifetime, efficiency, and electrocatalytic activity could potentially increase the process efficiency. Finally, scaling up the technology should be economically feasible. Flow technology and electrochemical reactor design are rarely evaluated in electrochemical lignin valorization. The batch process faces several drawbacks, for example, mixing difficulties and decreased volume-to-surface ratio when going from electrolyte to electrode especially when using large batch cells, therefore, it is recommended to move the technology to the flow process [117]. Already developed flow electrolyzers [118,119,120] can be used for scaling up the lignin electrolysis process technically.

In summary, electrocatalytic depolymerization of lignin is a promising technology for the production of industrial bio-based chemicals from biomass. A lot of advances have been made in recent years but still, a lot of challenges need to be addressed in order to use this technology industrially as well as to acquire the global cycle of carbon more sustainably.

## Data Availability

Any data or material can be made available by the corresponding author upon request.
